# Genetic Diversity, Carbapenem Resistance Genes, and Biofilm Formation in UPEC Isolated from Patients with Catheter-Associated Urinary Tract Infection in North of Iran

**DOI:** 10.1155/2022/9520362

**Published:** 2022-09-16

**Authors:** Sina Nasrollahian, Mehrdad Halaji, Akramasadat Hosseini, Mohammad Teimourian, Mojtaba Taghizadeh Armaki, Mehdi Rajabnia, Hemmat Gholinia, Abazar Pournajaf

**Affiliations:** ^1^Department of Medical Microbiology and Biotechnology, School of Medicine, Babol University of Medical Sciences, Babol, Iran; ^2^Infectious Diseases and Tropical Medicine Research Center, Health Research Institute, Babol University of Medical Sciences, Babol, Iran; ^3^Department of Pathology, School of Medicine, Babol University of Medical Sciences, Babol, Iran; ^4^Department of Urology, School of Medicine, Babol University of Medical Sciences, Babol, Iran; ^5^Social Determinants of Health Research Center, Health Research Institute, Babol University of Medical Sciences, Babol, Iran

## Abstract

**Background:**

Infections due to carbapenem-resistant *Enterobacteriaceae* (CRE) are associated in patients with urinary catheters alarming rate of emergency status. The aim of this study is to investigate the molecular causes of carbapenem resistance among UPEC as well as antimicrobial resistance trends. Additionally, the potential of isolates to produce biofilms, in addition to their clonal and genetic diversity, was investigated. *Material and Methods*. A cross-sectional study was accomplished on a collection of 76 non-duplicate UPEC isolates obtained from CAUTIs from May 2021 to September 2021. The modified carbapenem inactivation method (mCIM) and EDTA-modified carbapenem inactivation method (eCIM) test was performed for the detection of carbapenemase and metallo-beta-lactamase activity. Also, the presence of carbapenemase genes was determined using PCR assays. In 96-well microtiter plates, biofilm development was evaluated. ERIC-PCR was used to investigate the clonal and genetic variety of isolates.

**Results:**

A total of 76 confirmed UPEC isolates were obtained from patients mentioned to teaching hospitals in Babol, Iran. The results of antibiotic susceptibility testing revealed a high rate of antibiotic resistance against nalidixic acid (81.6%) and trimethoprim-sulfamethoxazole (80.3%). Among UPEC isolates, 63.2% and 13.2% of UPEC isolates were positive for MBL production. The frequencies of the studied genes are in order of *bla*_NDM_ (14.5%), *bla*_OXA-23_ (2.6%), and *bla*_OXA-48_ (2.6%). Forty-two isolates (55.3%) were positive for biofilm formation. ERIC-PCR revealed that UPEC isolates could be categorized into nine clusters A-I and five isolates were categorized as a singleton.

**Conclusion:**

The high prevalence of MDR and carbapenemase-producing isolates among the UPEC strain in this investigation is concerning. Moreover, the *bla*_NDM_ was the most frequent cause of producing metallo-beta-lactamase and carbapenemase. Also, analysis revealed a partial genetic similarity among the studied isolates, indicating that the same UPEC clones may have spread to other hospital units.

## 1. Introduction

Urinary tract infections (UTIs) are one of the most frequent bacterial illnesses, impacting 150 million people once a year around the world [1]. Almost less than 30% of women, who have a first episode of bacterial cystitis, will have a recurrent UTI within 6 months, with some having 6 or more infections in the year after the first episode. The urethra may be reinoculated with flora from the gastrointestinal tract, or a bladder epithelial reservoir may re-emerge, resulting in a recurrent UTI [1, 2].

The most common risk factor for complex UTI is indwelling urinary catheterization; catheter-associated UTIs (CAUTIs) account for 40% of all nosocomial infections worldwide and frequently lead to subsequent bloodstream infections [2]. Despite the fact that the risk of urine catheterization has been reduced as a result of increased awareness of the risk, a significant number of hospitalized patients still require urinary catheterization throughout their stay [3].

The uropathogenic *Escherichia coli* (UPEC) are strains of E. *coli* that diverge from their commensal position as bowel flora, develop and remain in the urine tract, and demonstrate a wide range of virulence characteristics and tactics that permit them to infect and originate illnesses in the urinary tract. These E. *coli* strains are known as UPEC because they are regularly linked to uropathogenic [4,5].

Extended-spectrum beta-lactamase (ESBL) generating organisms, carbapenem-resistant *Enterobacteriaceae* (CRE), and recently, colistin-resistant Gram-negative bacilli have all been linked to UTIs. CRE, which includes E. *coli, Klebsiella pneumoniae*, and *Enterobacter* spp., has become a significant worry for patients in hospitals [6].

CRE are multidrug-resistant Gram-negative bacteria that have developed resistance to carbapenems, a class of last-resort medicines. In *Enterobacteriaceae*, carbapenem resistance is mediated by a number of mechanisms, including the creation of efflux pumps, impermeability due to porin loss, and the expression of carbapenem-degrading-lactamases. Because of this, the World Health Organization has called CRE a critical disease that needs more research and the creation of new medicines [7–9].

The distinction between carbapenemase-producing carbapenem-resistant *Enterobacteriaceae* (CP-CRE) and non-CP-CRE is important for infection control and epidemiologic purposes because many carbapenemases are carried on mobile genetic elements that facilitate horizontal resistance transfer between Gram-negative organisms [10]; however, determining the mechanism of carbapenem resistance is not currently advised for treatment decision-making, and most clinical laboratories do not perform this routinely; though, this distinction between CP-CRE and non-CP-CRE is significant for infection control and epidemiologic purposes because of many carbapenem [11].

Numerous bacterial species generate exopolysaccharides (EPS), nucleic acid, and proteins to form biofilms, which are aggregations of bacterial populations inside an extracellular matrix [12, 13]. Biofilms allow bacterial colonies to cling to diverse inanimate and in-vivo settings, providing protection from harsh environmental conditions as well as toxic chemicals like antibiotics. Biofilm creation has been extensively researched in the pathogenesis of UTIs (particularly catheter-associated infections): this extracellular matrix enhances bacterial survival and chronicity by assisting in adhesion, providing protection against shear stresses in the urinary tract, and promoting bacterial survival and chronicity [14, 15].

Numerous studies have recently been conducted to determine the association between bacterial pathogen phenotypic features, biofilm development, and resistance to antibiotic. So, the purpose of this investigation was to discover the relationship between CRE and biofilm production in UPEC isolates and determine the genetic relationship between these strains in patients with urinary catheters [16]. Therefore, ERIC-PCR was used as a molecular method in this investigation into UPEC secluded patients who suffered from CAUTIs in the North of Iran.

## 2. Materials and Methods

### 2.1. Bacterial Isolates, Study Population, and Identification

A cross-sectional study was accomplished on a collection of 76 non-duplicate UPEC isolates gained from CAUTIs from May 2021 to September 2021. All patients were hospitalized in the North of Iran, Babol. The strains were isolated from pure cultures and identified in the laboratory of the Microbiology Research Center at the Hospital. UPEC strains were identified using the Gram-stain, the IMViC test, *β*-hemolytic activity, and other conventional biochemical tests. UPEC strains that were genetically confirmed as E. *coli* were kept in Brain Heart Infusion Broth (BHI) with 20% glycerol at −20°C.

### 2.2. Antimicrobial Susceptibility Testing

Antimicrobial resistance of UPEC isolates was investigated using disk diffusion on Mueller–Hinton agar plates (Merck, Germany) according to the Clinical and Laboratory Standards Institute guidelines. The antimicrobial agents tested were: cefotaxime (CTX), trimethoprim-sulfamethoxazole (SXT), ciprofloxacin (CP), ceftriaxone (CRO), nalidixic acid (NA), gentamicin (GM), amikacin (AN), nitrofurantoin (FM), and imipenem (IPM). E. *coli* ATCC® 25922™ was used for quality control [17].

### 2.3. Screening for Carbapenemase Production

The modified carbapenem inactivation method (mCIM) and EDTA-modified carbapenem inactivation method (eCIM) test was performed for the detection of carbapenemase and metallo-beta-lactamase activity in isolates [17].

### 2.4. Characterization of *β*-Lactamases at the Molecular Level

As previously disclosed, genomic DNA was isolated from fresh colonies [18]. The presence of carbapenemases genes including *bla*_OXA-48_, *bla*_OXA-23_*bla*_NDM_, *bla*_KPC_, *bla*_VIM_, and *bla*_IMP_, as previously described, PCR assays to determine were used. The PCR amplicons of UPEC isolates contain *bla*_IMP_, *bla*_NDM_, and *bla*_OXA-48_ genes were sequenced, and the DNA sequence of each gene was assigned in the GenBank nucleotide database at https://www.ncbi.nlm.nih.gov/blast/. The nucleotide sequences of the *bla*_IMP_, *bla*_NDM_, *bla*_OXA-48_, and *bla*_OXA-23_ genes have been assigned to GeneBank under the following accession numbers: ON817184, ON817185, ON817186, and OP235942.

### 2.5. Biofilm Formation by UPEC Isolates

The production of biofilms was measured in 96-well microtiter plates using the Stepanović et al. technique. In a nutshell, E. *coli* strains were cultured overnight and diluted to 0.5 McFarland turbidity. After that, a 1 : 100 dilutions of this suspension in new Tryptic soy broth (TSB) were produced, and 100 *μ*l of the diluted suspension was placed into each well of a microtiter plate, which was then incubated at 37°C for 48 hours. The connected cells were then fixed for 15 minutes in 200 *μ*l of 96% methanol (Merk, Germany). After that, 150 *μ*l of 2% crystal violet was added to each well, and the plates were incubated at room temperature for 15 minutes. Finally, each well received 150 *μ*l of 33% acetic acid, and the OD550 was determined using a microtiter-plate reader (Bio-Rad, USA). For the antibiofilm assay, the isolates with the highest biofilm-forming capacity were selected [19].

The ERIC-PCR method was employed to investigate all UPEC isolates, and the study's primer sequence was previously disclosed. Amplified products were assessed by electrophoresis through 1.5% agarose gels and DNA bands were visualized using ultraviolet light after staining with safe stain load dye (CinnaGen Co., Tehran, Iran). GelJ software was used to evaluate ERIC patterns, as previously stated. Isolates having a resemblance coefficient of 80% or higher were grouped together as the same genotypes.

### 2.6. Statistical Analysis

Differences in the frequency of resistance genes and antimicrobial resistance patterns between UPEC isolates were analyzed using the Chi-square test for each variable. A difference was considered statistically significant if the *P*value was less than 0.05.

## 3. Result

### 3.1. Bacterial Isolates and Antimicrobial Susceptibility Test

An entire 76 confirmed UPEC detaches were obtained from urine samples of patients mentioned to a teaching hospital in Babol, Iran. Among isolated samples, male and female frequencies were 35% (26/76) and 65% (50/76), respectively.

**Figure 1:**
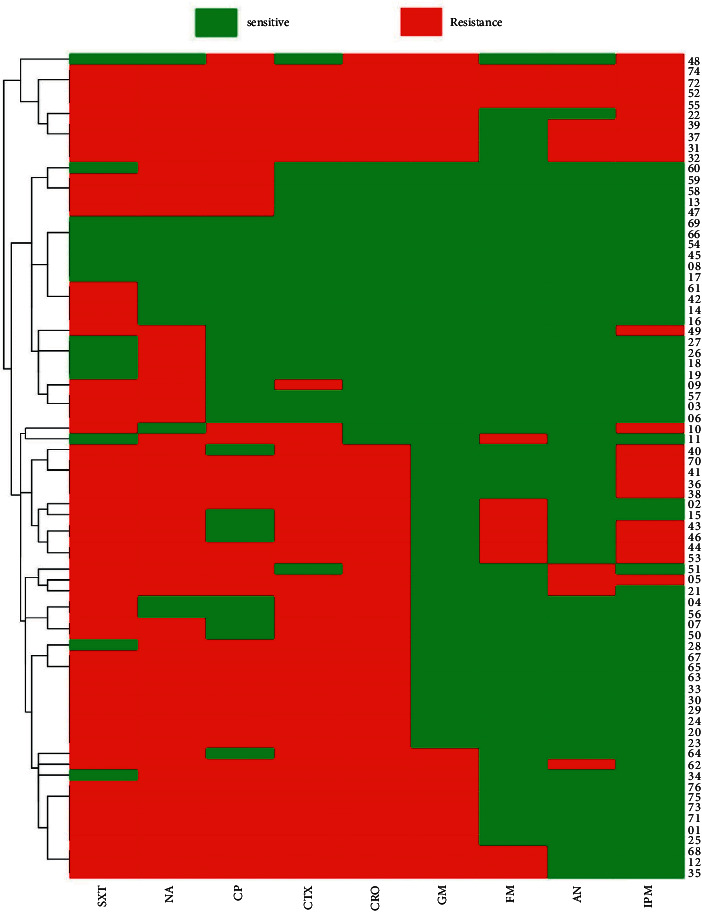
Heatmap and hierarchical clustering of UPEC isolates according to their antibiotic resistance profile of variables showing differences between isolates.

The results of antibiotic susceptibility testing for the 76 UPEC strains revealed a high rate of antibiotic resistance against nalidixic acid (81.6%) and trimethoprim-sulfamethoxazole (80.3%) as well as to cephalosporin (67.1% to cefotaxime and 65.8% to ceftriaxone). A high level of susceptibility was seen to amikacin (84.2%) and nitrofurantoin (81.6%). The results of antibiotic susceptibility testing for all isolates are presented in Figure 1.

### 3.2. Phenotypic Differentiation of MLBs and Class A KPC Carbapenemases

**Figure 2:**
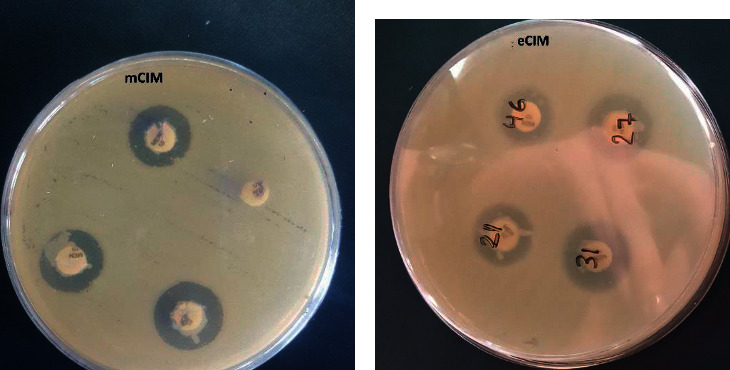
The results of mCIM and eCIM; (a) mCIM, (b) eCIM.

Carbapenemase test performed with the mCIM assay revealed that 63.2% (48/76) of UPEC isolates were positive for carbapenemase production. Moreover, phenotypic tests of mCIM and eCIM were used to detect and differentiate MBLs from serine carbapenemases. Among the UPEC isolates, 10 (13.2%) were positive for MBL production (Figure 2).

### 3.3. Carbapenemase Genes Detection

The frequencies of the studied genes are in order of *bla*_NDM_ (14.5%), *bla*_oxa-23_ (2.6%), *bla*_oxa-48_ (2.6%), *bla*_IPM_ (1.3%), *bla*_VIM_ (1.3%), and *bla*_KPC_ (0%), genes. Among the 48 carbapenemase-producing UPEC isolates, eight (16%) carried *bla*_NDM-1_, two (4%), and one isolate were positive for *bla*_OXA-48_ and *bla*_oxa-23_, respectively. The *bla*_NDM-1_ (10%) was the carbapenemase gene found in the most carbapenem-negative UPEC isolates. None of the carbapenem-negative UPEC strains carried *bla*_KPC_. Moreover, based on statical analysis, there is no relationship between the frequency of carbapenemase genes and CR-UPEC isolates. The frequency of carbapenemase encoding genes is presented in Tables 1 and 2.

### 3.4. Antibiotic Resistance Is Linked to Carbapenemase Production in UPEC

Resistance to CTX, CRO, and CP was found to be significantly associated with carbapenemase-producing isolates among the nine antibiotics tested. However, in comparison with carbapenemase–negative isolates, a high level of antibiotic-resistant was seen against IPM, AN, GM, SXT, and NA in carbapenemase-producing isolates.

### 3.5. Biofilm Formation

Forty-two (55.3%) of the 76 UPEC isolates tested positive for biofilm formation and were separated into three groups based on their capability to form biofilms. An entire of 34 (44.7%) of these isolates was classified as negative biofilm-producer isolates.

In addition, our investigation discovered a strong relationship between antimicrobial resistance patterns and weak and intermediated biofilm formation in distinct clusters, as shown in Table 1. No significant association was observed between biofilm production and antibiotic resistance. The antimicrobial resistance patterns of positive and negative biofilm formation isolates are shown in Table 3.

Positive biofilm formation isolates exhibited high levels of resistance to commonly used antibiotics especially NA (76%), SXT (71%), and CTX (88%), Table 2. Most of the positive biofilm formation isolates were MDR (resistance to three or more classes of antimicrobials) however, most of positive biofilm formations were susceptible to FM and AN. There was no significant relationship between antibiotic resistance and positive biofilm formation as compared to negative biofilm formation.

### 3.6. ERIC-PCR Results

**Figure 3:**
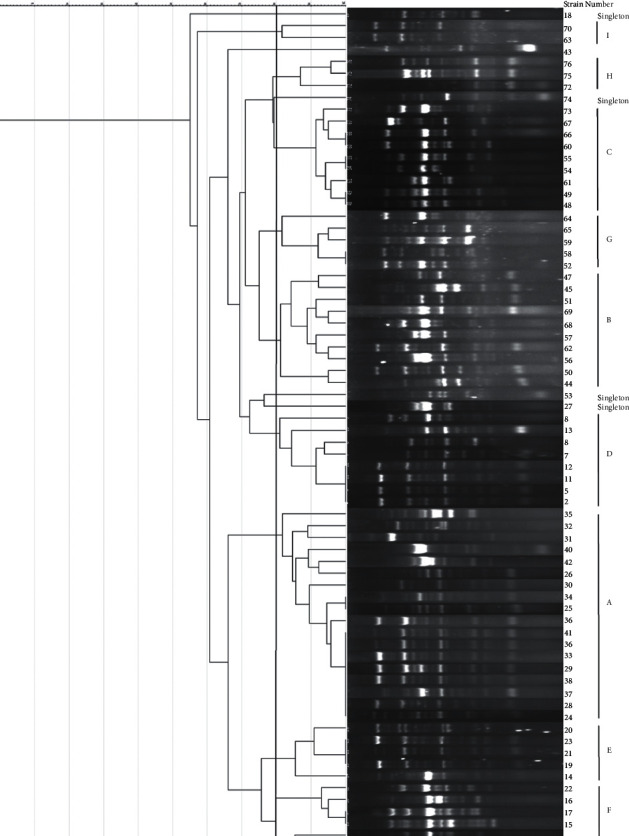
Enterobacterial repetitive intergenic consensus polymerase chain reaction (ERIC-PCR) profiles dendrogram of inpatients' uropathogenic *Escherichia coli* (UPEC) isolates.

Founded on a cut-off of 80% genetic similarity, ERIC-PCR discovered that 69 UPEC isolates could be categorized in nine clusters A (18 isolates), B (10 isolates), C (9 isolates), D (8 isolates), E (5 isolates), F (4 isolates), and G (5 isolates), H (3 isolates) , and I (2 isolates). Moreover, 5 isolates were categorized as singleton (Figure 3). Also, Table 4 present the details of carbapenemase-producing UPEC isolates.

## 4. Discussion

CAUTIs are the most frequent nosocomial infections, and they are linked to longer hospital stays, worse morbidity, and higher mortality [20]. Long-term urinary catheter use increases the risk of UTI, owing to bacteria's capacity to form a biofilm on the catheter that resists clearance by host defense and medications [21]. UPEC is the most common cause of UTIs, and the widespread use of antibiotics in human medicine for treatment, prevention, and prophylaxis has been linked to the rise of MDR strains [21, 22].

Because of their broad spectrum of antibacterial activity, carbapenems are usually utilized in experimental settings to treat MDR Gram-negative bacterial infections [23]. Still, some monitoring groups say that the overuse of antibiotics and the rise of organisms that are unaffected by carbapenems have become a major threat to global health [24].

So, figuring out the types of antibiotic resistance, how they spread, the characteristics of resistant bacteria, and the clonal relationships between isolates can help make treatment guidelines [25].

Thus, to the best of our knowledge, this is the first study to show the overall incidence of carbapenemase-related genes in recent UPEC isolates from Babol, Iran.

Our results showed that frequency of UTI in woman (65%) higher than in male (35%) because a combination of factors contributes to women being more susceptible. These factors such as length of urethra, more sensitive skin, placement of urethra, sexual contact, specific types of contraception, and pregnancy [26].

According to study by Zubair et al. frequency of UTI was 87.94% in female and 12.06% in male and another investigation by Magliano et al. showed frequency of UTI was highest in woman aged between 15 and 60 than in male [27, 28]. A ten-year surveillance study by Linhares et al. from the 155597 samples analyzed, UTI was more frequent in women (78.5%). Totally, E. *coli* was demonstrated in isolates from UTI patients in comparison with other bacteria like K. *pneumoniae* [29].

Particularly when it results from the empirical antimicrobial treatment of recurrent UTIs, UTIs are linked to high antibiotic use that has consequences for bacterial ecology and spreading of antibiotic resistance. Clinical issues, especially in women with recurrent UTIs, include the rise in MDR UPEC and antimicrobial resistance in UPEC. the rising incidence of MDR UPEC, particularly in developing nations [30]. In the present study, UPEC isolates showed the highest levels of resistance to sulfonamides 80.3%, quinolones 72.4%, and cephalosporin 66.4%, whereas aminoglycosides 77.6% and nitrofurans 62% were found to be the most effective antibiotics. Our results showed that resistance to nalidixic acid 81.6% was the highest. The most susceptible antibiotic was amikacin 84.2%.

A comparable form of susceptibility to antibiotics was described against uropathogenic E. *coli* isolates in previous studies from Iran [31–33], and Iraq [34]. Based on what we learned, nitrofurantoin and amikacin seem to be good antibiotics for treating UPEC-related UTIs.

Based on our findings, a high frequency of MDR isolates (76.3%) was observed for most of the antibiotics used against UTIs, which exceeded the previously displayed statistics in Spain(30%) [35], Iran (55.8%) [32], and Nepal (70.3%) [36].

These different results could be caused by a number of things, such as differences in the area covered by the study, differences in how antibiotics are prescribed, and the fact that some countries do not have a system for tracking antibiotic use [37].

In our investigation, we found that about 28.9% of UPEC isolates were resistant to one of the carbapenems tested.

Moreover, according to the results of phenotypic mCIM, 63.2% of isolates presented positive tests and produced phenotypically carbapenemase, which was more advanced than the rate reported in a study conducted by Jomehzadeh et al. Zowawi et al. [38, 39].

Moreover, a lesser commonness of carbapenemase-producing E. *coli* strains was described in Iran [32] and Egypt [40], in addition to in China [41].

Nonetheless, according to a meta-analysis study performed by Nasiri et al. the rates of CR in E. *coli* increased from 0.6% in 1997–2000 to 2.9% in 2013–2016 [42].

Longer hospitalizations and the usage of antibiotics may increase the risk of genetic variants and carbapenemase acquisition in hospitalized patients [43]. Furthermore, a rise in CRE prevalence may result in greater mortality, a longer stay in the hospital, and increased healthcare spending and utilization [44]. Moreover, our findings revealed that carbapenemase-producing UPEC were significantly resistant to cephalosporin and quinolones compared to noncarbapenemase-producers. Aminoglycosides are the most effective antibiotics against bacteria that are not able to produce carbapenemase. Although, the highest resistance to antibiotic in both groups to sulfonamides.

As previously stated, carbapenemase synthesis is one of the key mechanisms of carbapenem resistance in E. *coli* (i.e., NDM, KPC, VIM, IMP, and OXA) [7]. The mCIM consequences of the present study were further confirmed by PCR assay, and it was shown that *bla*_NDM_ (14.5%), *bla*_oxa-23_ (2.6%), and *bla*_OXA-48_ (2.6%), genes are predominantly found in the isolates and were responsible for resistance to carbapenem in UPEC.

Furthermore, according to statistical analysis, there is no relationship between the frequency of carbapenemase genes and CR-UPEC isolates. However, the *bla*_NDM_, *bla*_VIM_, *bla*_KPC_, *bla*_oxa-23_, and *bla*_OXA-48_ genes were not found in any isolates that were resistant to carbapenem.

Nasiri et al. found that the most commonly reported mechanisms of carbapenem resistance in E. *coli* were attributed to the presence of the *bla*_OXA-48_ (37.17%) and *bla*_NDM_ (21.92%) genes, respectively [42].

According to the Ambler classification system, OXA-48 is a b-lactamase that may hydrolyze penicillins and imipenem and has minimal activity against broad-spectrum cephalosporins. These bacteria can get the plasmid that has the *bla*_OXA-48_ gene on it and then make OXA-48 carbapenemase [45].

Several isolates were phenotypically carbapenemase-producing, similar to prior investigations, but carbapenemase genes were not discovered in any of the isolates. It is possible that the isolates' outer membranes have changed, or that AmpC b-lactamases have been overproduced [45].

It is possible that CRE is spreading over the world as a result of patient's traveling to other nations. Also, because CRE strains are resistant to many antimicrobial drugs, treatment options are limited. People, who have CRE, must be closely watched and given the right care [46].

In this investigation, 55.3% of UPEC isolates were able to produce biofilms in-vitro. Because UPEC likes to make biofilms, they can stay in the urinary system for longer. This could make UTIs worse, make them come back, and make it harder to treat them [22, 47].

In research undertaken in other nations and even different areas of Iran, the condition of UPEC biofilm production varies. In research by Soto et al., 46% of UPEC strains were found to be positive for in-vitro biofilm formation, whereas Rijavec et al. found 56% of UPEC strains to be positive [47, 48].

Behzadi et al. found that 47.6% of UPEC strains were moderate or strong biofilm producers, which agrees with our findings [49]. In a study conducted in Rasht, Iran, 94% of UPEC isolates were shown to be positive for biofilm formation in-vitro.

Another study by Nikzad et al. found that 85.8% of UPEC strains were both strong and weak biofilm makers [50]. Biofilm generation was estimated to be 62.5% in a study shown by Katongole et al. in 2020 on 200 UPEC isolates [51].

In this investigation, 48.4% of the isolates were strong biofilm producers, 15.6% were moderately potent, 21.8% were weak, and 14.2% did not create biofilms. These variances could be attributable to the genetic diversity of UPEC strains, and changes in frequency rates could also be influenced by the methodologies and culture medium employed, the kind of biofilm measurement method used, and the quantity and origin of the sample of examined E. *coli* isolates.

The presence of antibiotic resistance had no effect on UPEC isolates' ability to produce biofilms in-vitro. Biofilm-producing isolates, instead, had a greater rate of antibiotic resistance than nonbiofilm-producing isolates.

The phylogenetic dendrogram of ERIC-PCR showed that the 69 isolates can be differentiated into nine major clusters (A-I) with similarities ranging from 18 to 100%. Furthermore, ERIC-PCR dendrograms revealed a limited genetic similarity among the studied isolates, with just a few of them clustering into singleton types. This finding could point to a common source for inpatient UPEC isolates, as well as the proliferation of the same UPEC clones throughout hospital units.

Our findings are consistent with those of Mahmoud et al., who found that several UPEC isolates show identical ERIC-PCR patterns [52]. In a study conducted in Palestine by Adwan et al., all UPEC isolates had diverse ERIC-PCR profiles, with no identical banding patterns between them [53].

In conclusion, the high prevalence of MDR and carbapenemase-producing isolates among the UPEC strain in this investigation is concerning, and specialists must ensure that appropriate antibiotics are used at the necessary times and in adequate doses to prevent the formation of multidrug-resistant organisms. Moreover, the *bla*_NDM_ followed by *bla*_oxa-23_, were the furthermost frequent cause of producing MBL and carbapenemase. Furthermore, a high incidence of biofilm producer isolates, which was found in hospitalized patients, is a severe problem in this study, making UPEC infection treatment tough and complicated. Also, ERIC-PCR dendrogram analysis revealed a partial genetic similarity among the studied isolates, indicating that the same UPEC clones may have spread to other hospital units.

## Figures and Tables

**Table 1:** Carbapenemase production and its association with antibiotic resistance in UPEC.

Category	Antibiotics	Carbapenem-positive 48 no. (%)		Carbapenem-negative 10 no. (%)		Unknown-carbapenem 18 no. (%)		*P* value
		Resistant	Susceptible	Resistant	Susceptible	Resistant	Susceptible	
Carbapenem	IPM	14 (29)	34 (71)	2 (20)	8 (80)	6 (33)	12 (67)	0.756
Cephalosporin	CTX	34 (71)	14 (29)	2 (20)	8 (80)	15 (84)	3 (16)	0.002
	CRO	32 (66)	16 (44)	2 (20)	8 (80)	16 (89)	2 (11)	0.001

Aminoglycosides	AN	9 (19)	39 (81)	0	10 (100)	3 (16)	15 (84)	0.394
	GM	15 (31)	33 (69)	0	10 (100)	7 (38)	11 (62)	0.080

Sulfonamides	SXT	39 (81)	9 (19)	6 (60)	4 (40)	16 (89)	2 (11)	0.177
Quinolones	CP	32 (66)	16 (44)	2 (20)	8 (80)	14 (77)	4 (23)	0.007
	NA	41 (85)	7 (15)	6 (60)	4 (40)	15 (84)	3 (16)	0.165

Nitrofurans	FM	9 (19)	39 (81)	2 (20)	8 (80)	3 (16)	15 (84)	0.972
Genes	Genes	Positive	Negative	Positive	Negative	Positive	Negative	
	*bla *_NDM_	8 (16)	40 (84)	1 (10)	9 (90)	2 (11)	16 (89)	0.774
	*bla *_KPC_	0	48 (100)	0	10 (100)	0	18 (100)	0
	*bla *_oxa-48_	2 (4)	46 (96)	0	10 (100)	0	18 (100)	0.549
	*bla *_oxa-23_	1 (2)	47 (98)	0	10 (100)	1 (5.5)	17 (94.5)	0.629
	*bla *_IPM_	1 (2)	47 (98)	0	10 (100)	0	18 (100)	0.744
	*bla *_VIM_	1 (2)	47 (98)	0	10 (100)	0	18 (100)	0744

AN: Amikacin; IPM: Imipenem; FM: Nitrofurantoin; SXT: Trimethoprim-Sulfamethoxazole; CTX: Cefotaxime; GM: Gentamicin; CRO: Ceftriaxone; CP: Ciprofloxacin; and NA: Nalidixic acid.

**Table 2:** Distribution of metallo-beta-lactamase genes among UPEC isolates.

Genes	Metallo-beta-lactamase-positive 10		Metallo-beta-lactamase-negative 66		*P* value
	Positive no. (%)	Negative no. (%)	Positive no. (%)	Negative no. (%)	
*bla *_NDM_	1 (10)	9 (90)	10 (15)	56 (85)	0.666
*bla *_KPC_	10 (100)	0	66 (100)	0	—
*bla *_oxa-48_	0	10 (100)	2 (3)	64 (96)	0.577
*bla *_oxa-23_	0	10 (100)	2 (3)	64 (96)	0.577
*bla *_IPM_	0	10 (100)	1 (1.5)	65 (98.5)	0.695
*bla *_VIM_	0	10 (100)	1 (1.5)	65 (98.5)	0.695

**Table 3:** Biofilm formation and its association with antibiotic resistance in UPEC.

Category	Antibiotics	Biofilm-negative 34		Biofilm-weak and intermediate 42		*P* value
		Resistant no. (%)	Susceptible no. (%)	Resistant no. (%)	Susceptible no. (%)	
Cephalosporin	CTX	24 (70)	10 (30)	27 (64)	15 (36)	0.561
	CRO	24 (70)	10 (30)	26 (62)	16 (38)	0.428
Carbapenem	IPM	9 (26)	25 (74)	13 (30)	29 (70)	0.668
Sulfonamides	SXT	31 (91)	3 (9)	30 (71)	12 (29)	0.031
Quinolones	CP	23 (67)	11 (33)	25 (59)	17 (41)	0.465
	NA	30 (89)	4 (11)	32 (76)	10 (24)	0.178
Aminoglycosides	GM	9 (26)	25 (74)	13 (30)	29 (70)	0.668
	AN	4 (11)	30 (89)	8 (19)	34 (81)	0.387
Nitrofurans	FM	7 (20)	27 (80)	7 (17)	35 (83)	0.661
Genes	Genes	Positive	Negative	Positive	Negative	
	*bla *_NDM_	5(14)	29 (86)	6 (15)	36 (85)	0.959
	*bla *_KPC_	0	34 (100)	0	42 (100)	—
	*bla *_oxa-48_	2 (5)	32 (95)	0	42 (100)	0.111
	*bla *_oxa-23_	1 (3)	33 (97)	1 (2)	41 (98)	0.879
	*bla *_VIM_	1 (3)	33 (97)	0	42 (100)	0.263
	*bla *_IPM_	0	34 (100)	1 (2)	41 (98)	0.365

AN: Amikacin; IPM: Imipenem; FM: Nitrofurantoin; SXT: Trimethoprim-Sulfamethoxazole; CTX: Cefotaxime; GM: Gentamicin; CRO: Ceftriaxone; CP: Ciprofloxacin; and NA: Nalidixic acid.

**Table 4:** Characteristics of the carbapenemase-producing UPEC isolates.

Strain	Antibiotic resistance pattern	MBL-producing	Biofilm formation	Carbapenemase genes	ERIC type
2	FM, SXT, CTX, CRO, CP, NA	No	Weak		D
3	SXT, NA	Yes	Negative		
5	AN, IPM, SXT, CTX, CRO, CP, NA	Yes	Negative	*bla *_NDM_	D
7	SXT, CTX, CRO, NA	No	Negative		D
8		No	Weak		D
9	SXT, CTX, NA	No	Weak		D
10	IPM, SXT, CTX, CP	No	Weak		
11	FM, CTX, CP, NA	No	Weak		D
13	SXT, CP, NA	No	Weak		D
19	NA	No	Weak		E
23	SXT, CTX, CRO, CP, NA	No	Weak		E
24	SXT, CTX, CRO, CP, NA	No	Negative		A
25	SXT, CTX, GM, CRO, CP, NA	No	Negative	*bla *_oxa-23_	A
28	CTX, CRO, CP, NA	No	Intermediate		A
29	SXT, CTX, CRO, CP, NA	No	Negative		A
31	AN, IPM, SXT, CTX, GM, CRO, CP, NA	No	Weak	*bla *_NDM,_ *bla *_IPM_	A
34	CTX, GM, CRO, CP, NA	No	Weak		A
37	AN, IPM, SXT, CTX, GM, CRO, CP, NA	No	Intermediate	*bla *_NDM_	A
38	IPM, SXT, CTX, CRO, CP, NA	No	Intermediate		A
40	IPM, SXT, CTX, CRO, NA	Yes	Weak		A
41	IPM, SXT, CTX, CRO, CP, NA	No	Negative		A
46	IPM, FM, SXT, CTX, CRO, NA	No	Weak		
49	IPM, SXT, NA	No	Negative		C
50	SXT, CTX, CRO, NA	No	Weak		B
51	AN, SXT, CRO, CP, NA	No	Weak		B
52	AN, IPM, FM, SXT, CTX, GM, CRO, CP, NA	No	Negative	*bla *_NDM,_ *bla *_oxa-48_	G
53	IPM, FM, SXT, CTX, CRO, CP, NA	No	Negative	*bla *_NDM_	Singleton
54		No	Negative		C
55	AN, IPM, FM, SXT, CTX, GM, CRO, CP, NA	No	Weak	*bla *_NDM_	C
56	SXT, CTX, CRO	Yes	Weak		B
57	SXT, NA	No	Negative		
58	SXT, CP, NA	Yes	weak Weak		G
59	SXT, CP, NA	Yes	Intermediate		G
60	CP, NA	No	Negative		C
61	SXT	No	Negative		C
62	AN, SXT, CTX, GM, CRO, CP, NA	No	Negative		B
63	SXT, CTX, CRO, CP, NA	No	Negative		I
64	SXT, CTX, GM, CRO, NA	No	Weak		G
65	SXT, CTX, CRO, CP, NA	No	Weak		G
66		No	Weak		C
68	FM, SXT, CTX, GM, CRO, CP, NA	Yes	Negative		B
69		No	Negative		B
71	SXT, CTX, GM, CRO, CP, NA	Yes	Negative		
72	AN, IPM, FM, SXT, CTX, GM, CRO, CP, NA	No	Negative	*bla *_NDM,_ *bla *_oxa-48,_ *bla *_VIM_	H
73	SXT, CTX, GM, CRO, CP, NA	No	Negative		C
74	AN, IPM, FM, SXT, CTX, GM, CRO, CP, NA	No	Weak	*bla *_NDM_	Singleton
75	SXT, CTX, GM, CRO, CP, NA	Yes	Negative		H
76	SXT, CTX, GM, CRO, CP, NA	No	Negative		H

## Data Availability

Data are available on request from the authors.

## Abbreviation

UTIs: Urinary tract infections
CAUTIs: Catheter-associated UTIs
UPEC: The uropathogenic *Escherichia coli*
ESBL: Extended-spectrum beta-lactamase
CRE: Carbapenem-resistant *Enterobacteriaceae*
CP-CRE: Carbapenemase-producing carbapenem-resistant *Enterobacteriaceae*
EPS: Exopolysaccharides
BHI: Brain heart infusion broth
mCIM: The modified carbapenem inactivation method
eCIM: EDTA-modified carbapenem inactivation method.

## References

[1] Sharahi J. Y., Hashemi A., Ardebili A., Davoudabadi S. (2021). Molecular characteristics of antibiotic-resistant *Escherichia coli* and *Klebsiella pneumoniae* strains isolated from hospitalized patients in Tehran, Iran. *Annals of Clinical Microbiology and Antimicrobials*.

[2] Flores-Mireles A., Hreha T. N., Hunstad D. A. (2019). Pathophysiology, treatment, and prevention of catheter-associated urinary tract infection. *Topics in Spinal Cord Injury Rehabilitation*.

[3] Letica-Kriegel A. S., Salmasian H., Vawdrey D. K. (2019). Identifying the risk factors for catheter-associated urinary tract infections: a large cross-sectional study of six hospitals. *BMJ Open*.

[4] Wiles T. J., Kulesus R. R., Mulvey M. A. (2008). Origins and virulence mechanisms of uropathogenic *Escherichia coli*. *Experimental and Molecular Pathology*.

[5] Bien J., Sokolova O., Bozko P. (2012). Role of uropathogenic *Escherichia coli* virulence factors in development of urinary tract infection and kidney damage. *International Journal of Nephrology*.

[6] Halaji M., Shahidi S., Ataei B., Atapour A., Feizi A., Havaei S. A. (2021). Molecular epidemiology of blaCTX-M gene-producing uropathogenic *Escherichia coli* among Iranian kidney transplant patients: clonal dissemination of CC131 and CC10. *Annals of Clinical Microbiology and Antimicrobials*.

[7] Codjoe F. S., Donkor E. S. (2017). Carbapenem resistance: a review. *Medical Science*.

[8] Meletis G. (2016). Carbapenem resistance: overview of the problem and future perspectives. *Therapeutic Advances in Infectious Disease*.

[9] Kumudunie W. G. M., Wijesooriya L. I., Wijayasinghe Y. S. (2021). Comparison of four low-cost carbapenemase detection tests and a proposal of an algorithm for early detection of carbapenemase-producing Enterobacteriaceae in resource-limited settings. *PLoS One*.

[10] Pierce V. M., Simner P. J., Lonsway D. R., Das S. (2017). Modified carbapenem inactivation method for phenotypic detection of carbapenemase production among *Enterobacteriaceae*. *Journal of Clinical Microbiology*.

[11] Banerjee R., Humphries R. (2017). Clinical and laboratory considerations for the rapid detection of carbapenem-resistant *Enterobacteriaceae*. *Virulence*.

[12] Limoli D. H., Jones C. J., Wozniak D. J. (2015). Bacterial extracellular polysaccharides in biofilm formation and function. *Microbiology Spectrum*.

[13] Vu B., Chen M., Crawford R. J., Ivanova E. P. (2009). Bacterial extracellular polysaccharides involved in biofilm formation. *Molecules*.

[14] Trautner B. W., Darouiche R. O. (2004). Role of biofilm in catheter-associated urinary tract infection☆. *American Journal of Infection Control*.

[15] Andersen M. J., Fong C., La Bella A. A. (2022). Inhibiting host-protein deposition on urinary catheters reduces associated urinary tract infections. *eLife*.

[16] Donadu M. G., Mazzarello V., Cappuccinelli P. (2021). Relationship between the biofilm-forming capacity and antimicrobial resistance in clinical acinetobacter baumannii isolates: results from a laboratory-based in vitro study. *Microorganisms*.

[17] Wayne P. (2021). Clinical and laboratory standards institute: performance standards for antimicrobial susceptibility testing: twenty-fourth informational supplement, M100-S31. *Clinical and Laboratory Standards Institute (CLSI)*.

[18] Chen W. P., Kuo T. T. (1993). A simple and rapid method for the preparation of gram-negative bacterial genomic DNA. *Nucleic Acids Research*.

[19] S Stepanović, C Vuković, D Hola (2007). Quantification of biofilm in microtiter plates: overview of testing conditions and practical recommendations for assessment of biofilm production by staphylococci. *APMIS*.

[20] Jacobsen S. M., Stickler D. J., Mobley H. L., Shirtliff M. E. (2008). Complicated catheter-associated urinary tract infections due to *Escherichia coli* and *Proteus mirabilis*. *Clinical Microbiology Reviews*.

[21] McLellan L. K., Hunstad D. A. (2016). Urinary tract infection: pathogenesis and outlook. *Trends in Molecular Medicine*.

[22] Terlizzi M. E., Gribaudo G., Maffei M. E. (2017). UroPathogenic *Escherichia coli* (UPEC) infections: virulence factors, bladder responses, antibiotic, and non-antibiotic antimicrobial strategies. *Frontiers in Microbiology*.

[23] Doi Y. (2019). Treatment options for carbapenem-resistant gram-negative bacterial infections. *Clinical Infectious Diseases: An Official Publication of the Infectious Diseases Society of America*.

[24] Ventola C. L. (2015). The antibiotic resistance crisis: part 1: causes and threats. *P & T: A Peer-Reviewed Journal for Formulary Management*.

[25] Halaji M., Fayyazi A., Rajabnia M., Zare D., Pournajaf A., Ranjbar R. (2022). Phylogenetic group distribution of uropathogenic *Escherichia coli* and related antimicrobial resistance pattern: a meta-analysis and systematic review. *Frontiers in Cellular and Infection Microbiology*.

[26] Harrington R. D., Hooton T. M. (2000). Urinary tract infection risk factors and gender. *The Journal of Gender-Specific Medicine: JGSM: the Official Journal of the Partnership for Women’s Health at Columbia*.

[27] Zubair K. U., Shah A. H., Fawwad A., Sabir R., Butt A. (2019). Frequency of urinary tract infection and antibiotic sensitivity of uropathogens in patients with diabetes. *Pakistan Journal of Medical Sciences*.

[28] Magliano E., Grazioli V., Deflorio L., Leuci A. I., Mattina R., Romano P. (2012). Gender and age-dependent etiology of community-acquired urinary tract infections. *The Scientific World Journal*.

[29] Linhares I., Raposo T., Rodrigues A., Almeida A. (2013). Frequency and antimicrobial resistance patterns of bacteria implicated in community urinary tract infections: a ten-year surveillance study (2000-2009). *BMC Infectious Diseases*.

[30] Kot B. (2019). Antibiotic resistance among uropathogenic *Escherichia coli*. *Polish Journal of Microbiology*.

[31] Halaji M., Shahidi S., Atapour A., Ataei B., Feizi A., Havaei S. A. (2020). Characterization of extended-spectrum *β*-lactamase-producing uropathogenic *Escherichia coli* among iranian kidney transplant patients. *Infection and Drug Resistance*.

[32] Shahbazi S., Asadi Karam M. R., Habibi M., Talebi A., Bouzari S. (2018). Distribution of extended-spectrum *β*-lactam, quinolone and carbapenem resistance genes, and genetic diversity among uropathogenic *Escherichia coli* isolates in Tehran, Iran. *Journal of Global Antimicrobial Resistance*.

[33] Zangane Matin F., Rezatofighi S. E., Roayaei Ardakani M., Akhoond M. R., Mahmoodi F. (2021). Virulence characterization and clonal analysis of uropathogenic *Escherichia coli* metallo-beta-lactamase-producing isolates. *Annals of Clinical Microbiology and Antimicrobials*.

[34] Gatya Al-Mayahie S. M., Al-Guranie D. R. T., Hussein A. A., Bachai Z. A. (2022). Prevalence of common carbapenemase genes and multidrug resistance among uropathogenic *Escherichia coli* phylogroup B2 isolates from outpatients in wasit province/Iraq. *PLoS One*.

[35] García-Meniño I., Lumbreras P., Lestón L., Álvarez-Álvarez M., García V., Hammerl J. A. (2022). Occurrence and genomic characterization of clone ST1193 Clonotype 14-64 in uncomplicated urinary tract infections caused by *Escherichia coli* in Spain. *Microbiology Spectrum*.

[36] Gurung S., Kafle S., Dhungel B., Adhikari N., Thapa Shrestha U., Adhikari B. (2020). Detection of OXA-48 Gene in carbapenem-resistant *Escherichia coli* and *Klebsiella pneumoniae* from urine samples. *Infection and Drug Resistance*.

[37] Ghasemian A., Shafiei M., Hasanvand F., Shokouhi Mostafavi S. K. (2018). Carbapenem and colistin resistance in *Enterobacteriaceae*: worldwide spread and future perspectives. *Reviews in Medical Microbiology*.

[38] Jomehzadeh N., Jahangirimehr F., Chegeni S. A. (2022). Virulence-associated genes analysis of carbapenemase-producing *Escherichia coli* isolates. *PLoS One*.

[39] Zowawi H. M., Sartor A. L., Balkhy H. H., Walsh T. R., Al Johani S. M., AlJindan R. Y. (2014). Molecular characterization of carbapenemase-producing *Escherichia coli* and *Klebsiella pneumoniae* in the countries of the Gulf cooperation council: dominance of OXA-48 and NDM producers. *Antimicrobial Agents and Chemotherapy*.

[40] Abbas H. A., Kadry A. A., Shaker G. H., Goda R. M. (2019). Impact of specific inhibitors on metallo-*β*-carbapenemases detected in *Escherichia coli* and *Klebsiella pneumoniae* isolates. *Microbial Pathogenesis*.

[41] Tian X., Zheng X., Sun Y., Fang R., Zhang S., Zhang X. (2020). Molecular mechanisms and epidemiology of carbapenem-resistant *Escherichia coli* isolated from Chinese patients during 2002-2017. *Infection and Drug Resistance*.

[42] Nasiri M. J., Mirsaeidi M., Mousavi S. M. J., Arshadi M., Fardsanei F., Deihim B. (2020). Prevalence and mechanisms of carbapenem resistance in *Klebsiella pneumoniae* and *Escherichia coli*: a systematic review and meta-analysis of cross-sectional studies from Iran. *Microbial Drug Resistance*.

[43] Mariappan S., Sekar U., Kamalanathan A. (2017). Carbapenemase-producing *Enterobacteriaceae*: risk factors for infection and impact of resistance on outcomes. *International Journal of Applied and Basic Medical Research*.

[44] Livorsi D. J., Chorazy M. L., Schweizer M. L., Balkenende E. C., Blevins A. E., Nair R. (2018). A systematic review of the epidemiology of carbapenem-resistant *Enterobacteriaceae* in the United States. *Antimicrobial Resistance and Infection Control*.

[45] Hammoudi Halat D., Ayoub Moubareck C. (2020). The current burden of carbapenemases: review of significant properties and dissemination among gram-negative bacteria. *Antibiotics*.

[46] Iovleva A., Doi Y. (2017). Carbapenem-resistant *Enterobacteriaceae*. *Clinics in Laboratory Medicine*.

[47] Soto S. M. (2014). Importance of biofilms in urinary tract infections: new therapeutic approaches. *Advances in Biology*.

[48] Rijavec M., Müller-Premru M., Zakotnik B., Žgur-Bertok D. (2008). Virulence factors and biofilm production among *Escherichia coli* strains causing bacteraemia of urinary tract origin. *Journal of Medical Microbiology*.

[49] Behzadi P., Urbán E., Gajdács M. (2020). Association between biofilm-production and antibiotic resistance in uropathogenic *Escherichia coli* (UPEC): an in vitro study. *Diseases*.

[50] Nikzad M., Mirnejad R., Babapour E. (2021). Evaluation of antibiotic resistance and biofilm formation ability uropathogenic E. *coli* (UPEC) Isolated from pregnant women in Karaj. *Iranian Journal of Medical Microbiology*.

[51] Katongole P., Nalubega F., Florence N. C., Asiimwe B., Andia I. (2020). Biofilm formation, antimicrobial susceptibility and virulence genes of uropathogenic *Escherichia coli* isolated from clinical isolates in Uganda. *BMC Infectious Diseases*.

[52] Mahmoud A. T., Ibrahem R. A., Salim M. T., Gabr A., Halby H. M. (2020). Prevalence of some virulence factors and genotyping of hospital-acquired uropathogenic *Escherichia coli* isolates recovered from cancer patients. *Journal of Global Antimicrobial Resistance*.

[53] Adwan G., Issa B., Adwan K. (2015). Virulence profile, fluoroquinolone and quinolone resistance of uropathogenic *Escherichia coli* isolates recovered from thabet hospital-tulkarm, Palestine. *British Microbiology Research Journal*.

